# Is cervical screening preventing adenocarcinoma and adenosquamous carcinoma of the cervix?

**DOI:** 10.1002/ijc.30152

**Published:** 2016-05-06

**Authors:** Alejandra Castanon, Rebecca Landy, Peter D. Sasieni

**Affiliations:** ^1^ Centre for Cancer Prevention, Wolfson Institute of Preventive Medicine, Bart's and the London School of MedicineQueen Mary University of LondonCharterhouse SquareLondonUnited Kingdom

**Keywords:** cervical cancer, screening, adenocarcinoma, adenosquamous, effectiveness of screening

## Abstract

While the incidence of squamous carcinoma of the cervix has declined in countries with organised screening, adenocarcinoma has become more common. Cervical screening by cytology often fails to prevent adenocarcinoma. Using prospectively recorded cervical screening data in England and Wales, we conducted a population‐based case–control study to examine whether cervical screening leads to early diagnosis and down‐staging of adenocarcinoma. Conditional logistic regression modelling was carried out to provide odds ratios (ORs) and 95% confidence intervals (CIs) on 12,418 women with cervical cancer diagnosed between ages 30 and 69 and 24,453 age‐matched controls. Of women with adenocarcinoma of the cervix, 44.3% were up to date with screening and 14.6% were non‐attenders. The overall OR comparing women up to date with screening with non‐attenders was 0.46 (95% CI: 0.39–0.55) for adenocarcinoma. The odds were significantly decreased (OR: 0.22, 95% CI: 0.15–0.33) in up to date women with Stage 2 or worse adenocarcinoma, but not for women with Stage1A adenocarcinoma 0.71 (95% CI: 0.46–1.09). The odds of Stage 1A adenocarcinoma was double among lapsed attenders (OR: 2.35, 95% CI: 1.52–3.62) compared to non‐attenders. Relative to women with no negative cytology within 7 years of diagnosis, women with Stage1A adenocarcinoma were very unlikely to be detected within 3 years of a negative cytology test (OR: 0.08, 95% CI: 0.05–0.13); however, the odds doubled 3–5 years after a negative test (OR: 2.30, 95% CI: 1.67–3.18). ORs associated with up to date screening were smaller for squamous and adenosquamous cervical carcinoma. Although cytology screening is inefficient at preventing adenocarcinomas, invasive adenocarcinomas are detected earlier than they would be in the absence of screening, substantially preventing Stage 2 and worse adenocarcinomas.

## Abstract 2

**What's new?**

Organised cervical cancer screening programs have helped reduce the incidence of squamous carcinoma of the cervix, however the incidence of adenocarcinoma is on the rise. In some countries, this has led to a shift in the distribution of morphological types. The present study shows that, while cytology screening is capable of detecting early‐stage invasive adenocarcinoma and thereby prevents Stage 1B and worse adenocarcinomas, but it is substantially less good at detecting glandular than squamous precancerous lesions and therefore often fails to prevent stage 1A adenocarcinoma. The authors suggest that the effectiveness of cytology for cervical adenocarcinoma prevention may be strengthened when combined with other screening technologies.

The distribution of the main cervical cancer morphological types has changed considerably in countries with organised screening programmes.^1^, ^2^ Although squamous carcinoma continues to be the most frequent morphological type worldwide, increases in the incidence of adenocarcinoma (particularly among younger women) have been observed in several countries such as the United States,^3^^–^ 5 Italy^6^ and Sweden.^7^ In England the proportion of cervical cancers that are squamous carcinoma decreased from 82.6% in 1989 to 70.4% in 2009.^8^ Conversely, the proportion that are adenocarcinoma increased from 13.2% in 1989 to 22.1% in 2009, whereas the proportion that are adenosquamous carcinoma has remained fairly constant (2.3% vs. 3.6%).^8^

The first analysis providing evidence of the effectiveness of screening in the prevention of cervical cancer only considered squamous carcinoma.^9^ There has since emerged a growing body of literature suggesting that screening is less effective against adenocarcinoma than squamous carcinoma of the cervix.^7^, ^10^, ^11^, ^12^ The natural history of adenocarcinoma of the cervix differs from that of squamous carcinoma, with glandular precancerous lesions (adenocarcinoma *in situ*) thought to be harder to sample than squamous precancerous lesions (cervical intraepithelial neoplasia), as they typically develop within the endocervical canal, which often fails to be sampled by cytology.^13^ As a result, adenocarcinoma is often diagnosed at more advanced stages and has a worse prognosis than squamous carcinoma.^14^

Few authors have considered the effectiveness of screening against adenosquamous carcinoma, but it appears to be similar to that of squamous carcinoma.^15^

We hypothesise that cervical screening by cytology often fails to prevent adenocarcinoma of the cervix, but has the benefit of leading to early diagnosis and down‐staging. We update previous research,^15^ with data on an additional 9,472 women with cervical cancer, to explore the effect of screening on the likelihood of developing adenocarcinoma of the cervix in comparison to squamous carcinoma and adenosquamous carcinoma of the cervix.

## Material and Methods

### Study population

Currently, the NHS Cervical Screening Programme in England invites women aged 25–49 for three yearly screens, and women aged 50–64 for five yearly screens. In Wales, during the study period, women aged 25–64 were invited for three yearly screens. The programme is organised through a national call/recall system where those who test negative (and those who do not attend) are invited again after 3 or 5 years depending on age (routine recall action code), those with low‐grade abnormalities are triaged using HPV testing and those with high‐grade abnormalities are referred to colposcopy (suspended from recall). Prior to 2012, women with low‐grade abnormalities were invited for re‐screening at 6–12 months (early recall action code).

Since April 2007, data collection in England and Wales has been nationally audited, and it is estimated that 96% of cervical cancers in women aged 30–69 are included.^16^ Data on screening histories were abstracted from routinely recorded cervical cytology records held on the Cervical Screening Call/Recall System and were therefore not subject to recall bias. These records include all NHS (and many private provider) smears taken in the United Kingdom since 1988. After local NHS staff linked screening data to cases and controls, the data were anonymised locally before being transferred for cleaning and analysis. Guidelines on the collection of data for this audit and details of the design have been published previously.^17^, ^18^, ^19^

In our study, cases were women aged 30–69 when they were diagnosed with cervical cancer (ICD‐10 C53) in England (between January 1990 and July 2014) and Wales (between January 1999 and May 2013) (data were extracted from the database locked in July 2014), registered with an NHS general practitioner (GP). Eligible controls for each case were all other women registered with an NHS GP at the time of a case's diagnosis. Controls were assigned the date of diagnosis of their matched case. Two controls were individually matched within a year of birth to each case and on place of residence: one control had the same general practice as the case, and a second control had a different general practice but within the same administrative area. The second control was selected from a different GP to avoid possible overmatching if screening uptake was associated with the GP's enthusiasm for cervical screening. Controls were randomly selected (using a computer program) from women who satisfied the matching criteria. Data were collected on all selected controls, to eliminate possible participation bias. Stage was recorded as per the Fédération Internationale de Gynécologie et d'Obstétrique (FIGO) stages.

### Classification of screening exposure

To create a screening classification, women were defined as (*i*) never having been screened, (*ii*) up to date with their screening or (*iii*) having been screened in the past, but lapsed at the time of diagnosis. A woman was considered to be lapsed if the last smear had a routine action code (*i.e*. they were invited to return for screening at the normal interval, 3 or 5 years depending on age) dated over 3.5 (age <50) or 5.5 (age ≥50) years before diagnosis, had an early recall action code dated more than 1.25 years before diagnosis or had a suspend action code dated over 6 months before diagnosis. To be considered as “up to date with screening” in this article, women needed to have attended screening in the last 3.5 or 5.5 years depending on age. Women aged 66 and over needed to have been screened between the ages of 60–64 because screening is not offered beyond age 64.

The time since last negative screen was defined as the time between the last operationally negative smear and the date of diagnosis. An operationally negative smear has both a negative result and a routine action code, implying that the woman is not being followed‐up for a previous abnormal result. Women with no negative smears prior to diagnosis were combined with women whose last negative smear was >7 years before diagnosis.

### Statistical analysis

Conditional logistic regression modelling was carried out to provide odds ratios and 95% confidence intervals (CIs).

To explore whether the differences in the effect of screening were due to differences in the age distributions for each morphological type of cancer, we carried out an (internally) age‐standardised analysis of squamous carcinomas as follows: for each woman with adenocarcinoma, we randomly selected two age‐matched (within 24 months of age at diagnosis) women with squamous carcinoma. Analysis was then restricted to those age‐matched squamous cases and their controls, thus giving the squamous carcinoma cases the same age distribution as the adenocarcinomas.

Women were excluded if the morphological type of cancer was recorded as other (*N* = 330) or unknown morphology in the dataset (*N* = 1,347).

Analyses were carried out in Stata version 12.^20^

## Results

The analysis included 12,418 women with cervical cancer diagnosed aged 30–69 and 24,453 controls. Of the cancers in our study, 9,472 (76.3%) have not been analysed before; the remaining 23.7% were included in a previous publication.^15^ The majority of the cancers were squamous carcinoma (74.7%), followed by adenocarcinoma (21.9%) and adenosquamous carcinoma (3.4%). Among those with known stage, a large proportion of squamous carcinoma were diagnosed as Stage 1A cancer (38.8%) compared to adenocarcinoma (24.2%) and adenosquamous carcinoma (6.9%) (Table 1). However, 53.4% of adenocarcinoma (and 55.6% of adenosquamous) cancers were diagnosed as Stage 1B compared to 31.2% of squamous. Over a third of adenosquamous carcinoma was diagnosed as Stage 2 or worse (37.5%), compared to less than a third of squamous and adenocarcinoma (30 and 22.4% respectively). Of particular note is the very low proportion (7%) of adenosquamous carcinoma that were diagnosed at Stage 1A compared to 39% of squamous cases and 24% of adenocarcinomas.

**Table 1:** Main characteristics of women included in the study

	**Squamous**		**Adenocarcinoma**		**Adenosquamous**	
	***N***	**%**	***N***	**%**	***N***	**%**
**FIGO stage**						
1A	3,051	38.8	537	24.2	24	6.9
1B	2,449	31.2	1,183	53.4	194	55.6
2+	2,355	30.0	496	22.4	131	37.5
Not recorded	1,419	–	501	–	78	–
**Age group**						
30–39	3,640	39.2	1,008	37.1	148	34.7
40–49	2,621	28.3	903	33.2	125	29.3
50–69	3,013	32.5	806	29.7	154	36.1
**Screening history**						
Screened up to date	2,335	25.2	1,203	44.3	128	30.0
Screened lapsed	4,310	46.5	1,118	41.1	187	43.8
Never screened	2,629	28.3	396	14.6	112	26.2
Total	9,274	100	2,717	100	427	100

Of women with adenocarcinoma of the cervix, 44.3% were up to date with their screening, and the proportion of women with adenocarcinoma who had never been screened (14.6%) was lower than the proportion observed for the two other histological types (28.3% for squamous and 26.2% for adenosquamous) (Table 1). The odds ratio of developing Stage 1A adenocarcinoma was not significantly lower in women with up to date screening compared to never screened women (OR: 0.71, 95% CI: 0.46–1.09) (Table 2). The odds ratio was double among women with lapsed screening compared to those never screened (OR: 2.35, 95% CI: 1.52–3.62). Conversely, the odds ratio of developing Stage 1B (OR: 0.54, 95% CI: 0.41–0.71) or Stage 2 or worse (OR: 0.22, 95% CI: 0.15–0.33) adenocarcinoma of the cervix was significantly decreased in women with up to date screening compared to those never screened. Although the odds ratio of developing Stage 1A squamous carcinoma was increased in lapsed attenders (OR: 1.31, 95% CI: 1.13–1.52) compared to never screened women, it was significantly decreased in both up to date and lapsed attenders with Stage 2 or worse cancer (Table 2). The odds of a diagnosis of adenosquamous cancer, both overall and by stage, were very similar to the odds observed for squamous carcinoma, while those for adenocarcinoma were always higher.

**Table 2:** Odds ratios (ORs) and 95% confidence intervals (CIs) of developing cervical cancer by morphological type, screening classification and stage

	**Age 30–69**							
**All FIGO stages**	**Squamous**		**Adenocarcinoma**		**Adenosquamous**		**Age‐standardised squamous cancer** a	
	**OR**	**95% CI**	**OR**	**95% CI**	**OR**	**95% CI**	**OR**	**95% CI**
Screened up to date	0.14	(0.12–0.15)	0.46	(0.39–0.55)	0.16	(0.11–0.24)	0.13	(0.12–0.15)
Screened lapsed	0.76	(0.70–0.83)	1.28	(1.07–1.53)	0.70	(0.47–1.04)	0.73	(0.66–0.82)
Never	1		1		1		1	
**Stage 1A**								
Screened up to date	0.27	(0.23–0.31)	0.71	(0.46–1.09)	0.27	(0.04–1.77)	0.25	(0.20–0.30)
Screened lapsed	1.31	(1.13–1.52)	2.35	(1.52–3.62)	1.12	(0.16–7.90)	1.20	(0.99–1.46)
Never	1		1				1	
**Stage 1B**								
Screened up to date	0.17	(0.14–0.20)	0.54	(0.41–0.71)	0.24	(0.13–0.44)	0.17	(0.13–0.21)
Screened lapsed	0.93	(0.78–1.10)	1.43	(1.07–1.91)	0.81	(0.43–1.53)	0.87	(0.69–1.09)
Never	1		1		1		1	
**Stage 2+**								
Screened up to date	0.05	(0.04–0.06)	0.22	(0.15–0.33)	0.07	(0.03–0.15)	0.07	(0.06–0.09)
Screened lapsed	0.40	(0.33–0.47)	0.59	(0.38–0.90)	0.58	(0.28–1.18)	0.51	(0.43–0.61)
Never	1		1		1			

a Two squamous cancers were selected matching on age to each adenocarcinoma, so as to give the squamous cancers the same age distribution as the adenocarcinomas.

To ensure that the differences in the effect of screening were not due to differences in the age distributions for each morphological type of cancer, we estimated odds ratios for a restricted set of women with squamous cancers with the same age distribution as women with adenocarcinoma (Table 2). These age‐standardised estimates were similar to those that included all squamous cancers, showing that the difference in results between squamous carcinoma and adenocarcinoma was not a result of differences in age distribution.

Compared to never screened women, adenocarcinoma Stage 1A was very unlikely to be detected in women within 3 years of a negative cytology test (OR: 0.08, 95% CI: 0.05–0.13); however, the odds of Stage 1A adenocarcinoma was double at 3–5 years after a negative test (OR: 2.30, 95% CI: 1.67–3.18) (Table 3). Similarly, the risk of developing Stage 1B adenocarcinoma within 3 years of a negative cytology test was small (OR: 0.19, 95% CI: 0.15–0.23) compared to never screened women, but an increased risk was observed for women whose last negative screen was 3–5 years earlier (OR: 1.32, 95% CI: 1.07–1.62). The odds of developing squamous carcinoma remained low up to 7 years after a negative test. Again, the magnitude of the effect observed for adenosquamous cancer was similar to that for squamous cancer, while that for adenocarcinoma was always higher.

**Table 3:** Odds ratio (OR) and 95% confidence intervals (CIs) of developing cervical cancer by morphology, time since last negative test and stage

	**Squamous**		**Adenocarcinoma**		**Adenosquamous**	
	**OR**	**95% CI**	**OR**	**95% CI**	**OR**	**95% CI**
**Stage 1A**						
<3 years	0.02	(0.02–0.03)	0.08	(0.05–0.13)	0.25	(0.00–0.29)
3–5 years	0.88	(0.77–1.01)	2.30	(1.67–3.18)	0.28	(0.05–1.66)
5–7 years	1.09	(0.91–1.30)	2.76	(1.74–4.37)	0.55	(0.11–2.74)
>7 years or never	1		1		1^a^	
**Stage 1B**						
<3 years	0.05	(0.04–0.06)	0.19	(0.15–0.23)	0.09	(0.05–0.17)
3–5 years	0.51	(0.43–0.59)	1.32	(1.07–1.62)	0.78	(0.47–1.29)
5–7 years	0.55	(0.45–0.68)	1.23	(0.93–1.62)	1.45	(0.69–3.04)
>7 years or never	1		1		1	
**Stage 2**+						
<3 years	0.04	(0.03–0.05)	0.17	(0.13–0.24)	0.07	(0.03–0.14)
3–5 years	0.14	(0.11–0.17)	0.43	(0.31–0.60)	0.09	(0.04–0.23)
5–7 years	0.20	(0.15–0.25)	0.52	(0.33–0.80)	0.34	(0.15–0.81)
>7 years or never	1		1		1	

a There are only 24 women with adenosquamous carcinoma Stage 1A.

## Discussion

We have shown for the first time that screening is associated with a decreased odds of advanced‐stage adenocarcinoma of the cervix and an increased odds of early‐stage adenocarcinoma. Despite over 40% of women diagnosed with adenocarcinoma of the cervix having an up to date screening history, no reduction in the odds of developing Stage 1A adenocarcinoma was observed. However, Stage 1A adenocarcinoma was very unlikely to be diagnosed within 3 years of a negative screen, and a significant reduction in the odds of adenocarcinoma was observed with up to date screening for cancers Stage 1B or worse. This suggests that, although cytology may lack sensitivity for the detection of precursors of adenocarcinoma, it is sensitive at detecting early‐stage adenocarcinoma. The odds of developing Stage 1B or worse cancers were reduced in women with up to date screening. The effect of screening on adenosquamous carcinoma was similar to that observed for squamous carcinoma, but the magnitude of the effect was smaller.

A strength of the present study is its use of routinely recorded data on screening, linked to cancer diagnosis in a case–control design that avoids selection and recall bias. To our knowledge, this is the largest study assessing the impact of screening on adenocarcinomas of the cervix. Our study includes 2,717 women (of whom 2,216 have known FIGO stage) diagnosed with adenocarcinoma, compared to a combined total of 1,177 women from seven other studies.^7^, ^10^, ^11^, ^12^, ^21^, ^22^, ^23^

It is well documented that screening is less effective in young women,^24^ and there was concern that the difference in the effect of screening between morphological types could be due to a difference in the age distribution of women with different histological types of cervical cancer.^10^ We found that, after internal age‐standardisation, the odds ratio of developing squamous cervical cancer did not significantly change.

This is an observational study, so we cannot prove that the association between screening and a reduction in the risk of developing cervical cancer is causal. However, there is increasing evidence,^9^ including a randomised controlled trial,^25^ suggesting the association is indeed causal. We have no information on risk factors for cervical cancer (such as smoking and sexual history); however, the likelihood that the difference in the odds ratios is due to confounding seems small, because the known risk factors for squamous and adenocarcinoma of the cervix are similar.^26^

Our results are in line with the rest of the literature. Several authors have found cytology screening to be less effective in preventing adenocarcinoma than squamous carcinoma of the cervix.^10^, ^11^ Only one small study, including 17 adenocarcinomas, found no difference in the effect of screening between morphological types.^21^ Mitchell *et al*.^12^ showed a reduction in the risk of adenocarcinoma within a year of a negative test, and Nieminen *et al*.^27^ showed that mortality among women with adenocarcinoma of the cervix decreased, while incidence remained unchanged, both suggesting but not directly showing that screening is down‐staging adenocarcinoma. More directly, Pettersson *et al*.^7^ showed a 5‐fold increase in the proportion of cancers being diagnosed as adenocarcinoma, particularly among young women, but also showed that they are being diagnosed at an earlier stage in the latest screening cohorts. Further, Andrae *et al*.^28^ showed that survival from adenocarcinoma of the cervix (regardless of the stage) was better among those who had been screen‐detected compared to those with symptomatic presentation. However, no difference was observed (after adjusting for stage) between those who were up to date with their screening and those who had not been screened or were lapsed.

Given the rarity of diagnoses of adenocarcinoma *in situ* or high‐grade cervical glandular intraepithelial neoplasia, it is not surprising that the majority of adenocarcinomas are not prevented by screening. However, there is evidence of a positive association between screening coverage and the proportion of adenocarcinoma diagnosed.^13^ The low risk of Stage 2 or worse adenocarcinoma within 3 years of a negative cervical cytology, and at 3–7 years after a previous negative, suggests that cytology is good at detecting early‐stage invasive adenocarcinoma, and this is primarily leading to the reduction in advanced adenocarcinoma associated with regular screening.

The results suggest that screening is inefficient at preventing adenocarcinoma of the cervix, but is good at detecting Stage 1A adenocarcinoma. In the absence of screening, adenocarcinoma typically will not be diagnosed until Stage 1B or worse. Thus, Stage 1A adenocarcinoma is most common in women screened irregularly, in whom there is sufficient time for a Stage 1A cancer to have developed since their last screen, but insufficient time for it to have progressed to Stage 1B or worse.

The fact that screening does have an effect on Stage 1B or worse adenosquamous carcinoma suggests that the squamous component must be stronger and/or appear earlier in the carcinogenic process, allowing its detection through screening. There is only sparse literature on the epidemiology of cervical adenosquamous carcinoma, but based on the mix of HPV types found in these cancers (HPV‐16 (39%), −18 (32%), −45 (12%), −31 (2%) and −33 (3%)^29^) it could be assumed that they have more in common with adenocarcinoma than with squamous carcinoma. This is at odds with the findings here, unless adenosquamous cancers develop primarily from HPV18 positive squamous carcinoma.

It has been suggested that in cytology the lack of sensitivity to adenocarcinoma may be due to these cancers developing in the endocervical canal, making sampling of abnormal cells difficult. The introduction of primary HPV testing may help tilt the balance in favour of adenocarcinoma. A pooled analysis from the HPV primary screening trials examining cervical cancer suggests that HPV testing will have a bigger impact on adenocarcinoma (OR: 0.31, 95% CI: 0.14–0.69) than on squamous carcinoma (OR: 0.78, 95% CI: 0.49–1.25) compared to cytology.^30^ However, it is unclear whether HPV testing is more or less sensitive than cytology in detecting early‐stage invasive adenocarcinoma.

Five high risk HPV types are found in 92% of adenocarcinomas (HPV‐16 (41.6%), −18 (38.7%), −45 (7.0%), −31 (2.2%) and −33 (2.1%)).^13^, ^29^ HPV vaccination will provide the best prevention strategy against adenocarcinomas of the cervix. Both available vaccines protect against HPV types 16 and 18, and the bivalent vaccine (Cervarix) has strong cross‐protection against types 45, 31 and 33.^31^ Nevertheless, there is some evidence to suggest that, even after testing with multiple HPV assays, some cancers remain HPV negative, and these are more likely to be adenocarcinomas.^32^

## Conclusions

Cytology based screening has been less effective in preventing adenocarcinoma of the cervix than in preventing squamous carcinoma. However, screening detects adenocarcinoma earlier than diagnosis in the absence of screening, leading to a down‐staging of disease. In the future, a combination of HPV vaccination, HPV testing and new technologies will result in a considerable decrease in the burden of adenocarcinoma of the cervix.

## Authors’ Contributions

P.D.S. had full access to all of the data in the study and confirms that it is an honest, accurate and transparent account of the study being reported, that no important aspects of the study have been omitted; and that any discrepancies are disclosed. P.D.S. conceived and designed the study. A.C. analysed the data and wrote the first draft of the manuscript. R.L analysed the data. All authors commented on and approved the final draft of the manuscript.
